# Local Therapies for Hepatocellular Carcinoma and Role of MRI-Guided Adaptive Radiation Therapy

**DOI:** 10.3390/jcm12103517

**Published:** 2023-05-17

**Authors:** Yirong Liu, Brian Chou, Amulya Yalamanchili, Sara N. Lim, Laura A. Dawson, Tarita O. Thomas

**Affiliations:** 1 Department of Radiation Oncology, Northwestern Medicine, Chicago, IL 60611, USA; 2 Department of Radiation Oncology, Loyola University Medical Center, Maywood, IL 60153, USA; 3 Department of Radiation Oncology, Princess Margaret Cancer Centre, University of Toronto, Toronto, ON M5S 1A1, Canada

**Keywords:** hepatocellular carcinoma (HCC), liver-directed therapies (LDTs), stereotactic ablative body radiation (SABR), transarterial radioembolization (TARE), MRI-guided SABR, stereotactic MRI-guided on-table adaptive radiotherapy (SMART)

## Abstract

Hepatocellular carcinoma (HCC) is the most common liver tumor, with a continually rising incidence. The curative treatment for HCC is surgical resection or liver transplantation; however, only a small portion of patients are eligible due to local tumor burden or underlying liver dysfunction. Most HCC patients receive nonsurgical liver-directed therapies (LDTs), including thermal ablation, transarterial chemoembolization (TACE), transarterial radioembolization (TARE), and external beam radiation therapy (EBRT). Stereotactic ablative body radiation (SABR) is a specific type of EBRT that can precisely deliver a high dose of radiation to ablate tumor cells using a small number of treatments (or fractions, typically 5 or less). With onboard MRI imaging, MRI-guided SABR can improve therapeutic dose while minimizing normal tissue exposure. In the current review, we discuss different LDTs and compare them with EBRT, specifically SABR. The emerging MRI-guided adaptive radiation therapy has been reviewed, highlighting its advantages and potential role in HCC management.

## 1. Introduction

Hepatocellular carcinoma (HCC) is the most common liver tumor (comprising 75–85% of cases), which is the sixth most common cancer and the third most common cause of cancer-related death worldwide [1,2]. The disease burden of HCC in the US has been continually growing in the past decades, with incidence rates tripling since 1980 [2,3,4,5,6,7]. HCC is now the fifth leading cause of cancer-related death in the US [6,7,8]. The widely accepted risk factors for HCC differ across continents [9,10,11]. In East Asian countries, HCC is tightly linked with chronic hepatitis and aflatoxin exposure [3,6,11], while in the US, HCC is more related to nonalcoholic fatty liver disease as well as alcoholic cirrhosis [6,12,13]. For patients with high-risk factors for HCC, including cirrhosis from any cause, chronic hepatitis B infection, or hereditary hemochromatosis, experts may recommend screening with alpha-fetoprotein (AFP) and liver ultrasound [14,15,16,17]. However, these methods are used on an individualized basis. Most HCC patients are diagnosed with advanced-stage disease, with less than 30% diagnosed with very early or early-stage disease [16,18].

The current curative treatment for HCC patients is surgical resection, thermal ablation, or liver transplant [17]. However, only 15–30% of newly diagnosed HCC patients are eligible for surgical resection due to local tumor burden or underlying liver dysfunction. The rest of HCC patients, who are not candidates for the surgical approach, are usually recommended for nonsurgical liver-directed therapies (LDTs). Several societies have published their guidelines regarding HCC management, including the Barcelona Clinic Liver Cancer (BCLC) strategy, the National Comprehensive Cancer Network (NCCN) hepatobiliary guidelines, and the American Society for Radiation Oncology (ASTRO) guidelines [19,20,21]. The BCLC strategy is the most widely used management guideline [17]. The BCLC system stages HCC patients according to tumor burden, liver function, and physical status and offers treatment recommendations for each stage. In its latest version, the local treatment options include TACE, TARE, resection, ablation, and transplant. In the NCCN hepatobiliary cancer guideline, HCC patients are recommended for surgical resection if feasible, while other available LDTs, including ablation, arterially directed therapy, and radiation therapy [20,22], are discussed. In early 2022, the ASTRO published its first practice guideline for liver cancer management. Radiation therapy is recommended for several clinical scenarios, including bridging therapy to transplant, local control (LC) for non-ablation candidates, and patients with macrovascular invasion (MVI) [21]. Each LDT has its advantages and limitations. In the current review, we discuss each LDT for HCC, focusing on the comparison of RT with other modalities. We also discuss a new local therapy option with radiation, i.e., MR-guided adaptive radiation therapy (MRgRT). Its advantages and potential role in HCC management are highlighted.

## 2. Liver-Directed Therapies

### 2.1. Thermal Ablation

Thermal ablation strategies for localized hepatocellular carcinoma seek to cure those patients with small HCC (<2 cm). It is also an alternative for those patients who are not candidates for resection or transplant and have up to three HCC lesions, each smaller than 3 cm. Over the last four decades, there have been a variety of direct applicator methods introduced capable of selective tumor ablation using a direct percutaneous interstitial applicator, though these methods are not exclusive to liver-directed therapy. 

Currently, there are two major thermal ablation techniques commonly implemented in the United States: radiofrequency ablation (RFA) and microwave ablation (MWA). Older techniques for ablation include interstitial laser ablation and interstitial cryotherapy [23]. Historically, laser ablation uses fiber-optic cables or needles inserted into the tumor to deliver high-intensity light, which converts to heat within tissues and yields tumor necrosis. Cryoablation involves the use of a freezing agent (super-cooled gases or liquids) circulated into a probe tip that is inserted into the tumor and causes rapid freezing and thawing, thereby provoking cell membrane destruction. Laser ablation and cryoablation are not discussed further in this review as they are no longer routinely used in the United States. 

#### 2.1.1. Radiofrequency Ablation (RFA)

Approved by the Food and Drug Administration (FDA) in 1997, the use of radiofrequency energy for tissue ablation was rapidly adopted as an alternative to surgical resection of hepatocellular cancers and, to date, is the most reported of the aforementioned thermal ablation techniques. Electrodes are inserted into the tumor, and rapidly alternating electrical currents (~500 kHz) run through the electrode tip, causing rapid heating of the electrode and surrounding tissues. Access to the tumor can be achieved percutaneously or via laparoscopic surgical techniques, or less commonly, using open surgical techniques such as a laparotomy. In order to modulate the size of the ablative sphere, catheters may be fitted with multiple “prongs”, or tines, which expand radially outward to ablate larger lesions [23]. Most of the new devices now use a single linear applicator. For tumors less than 5 cm in diameter, recurrence-free survival rates approach 70–80% at 2 years [24,25]. Small prospective randomized studies of ablation versus resection for smaller tumors (<3–5 cm) demonstrated similar rates of disease control and overall survival [24,25]. A prospective study of 50 consecutive patients undergoing RFA found that the rate of complete tumor necrosis dropped to less than 30% for tumors ≥3 cm but exceeded 60% for tumors <3 cm [26]. Another study was able to demonstrate a complete necrosis rate exceeding 95% for very small tumors <2 cm in diameter undergoing RFA [27]. While ablation success rates remain relatively high, data regarding survival are mixed, as mortality is heavily driven by the degree of cirrhosis (i.e., Child–Pugh class), tumor size, and the number of tumors, and this remains the case for all liver-directed bridging therapies discussed in this review. 

#### 2.1.2. Microwave Ablation (MWA)

Similar to RFA, microwave ablation also uses an interstitial source (probe) inserted into the tumor for thermal ablation. An antenna probe, connected to a microwave power supply, emits microwaves (~2450 MHz and akin to a kitchen microwave frequency), which are absorbed by water molecules, resulting in the heating of hydrated tissues [23]. In recent decades, the use of MWA has become more common in Asian and European countries, while those in the United States have relied more on RFA for thermoablation. Available randomized data suggests they may be somewhat interchangeable options, as they appear to have similar efficacy and complication rates [28,29].

Important considerations for thermoablation include a patient’s eligibility for invasive procedures and the accessibility of the lesion epicenter by the operator. The heat sink effect is one of the most important limitations of thermal ablation, which is usually more significant in RFA. As to MWA, the heat sink effect is less concerning since it can heat rapidly. In the current era, RFA and MWA remain reasonable ablative options for smaller, usually peripheral targets, typically 3 cm or less.

Advances in interstitial ablation may be on the horizon. Irreversible electroporation (IRE) is a relatively new technique that uses non-thermal electrical pulses to cause cellular membrane disruption and apoptosis and is not limited by the heat sink effect of adjacent vasculature [30,31]. Future studies may further establish its utility for hepatocellular carcinoma, but currently, IRE is not regarded as a standard of care treatment. 

### 2.2. Transarterial Chemoembolization (TACE)

The introduction of transarterial embolization (TAE) techniques for HCC initially sought to produce tumor necrosis via vascular ischemia. To this end, a variety of particles/microspheres have been used to occlude the arterial blood supply to the HCC tumor by direct injection into the hepatic arterial system, typically by an interventional radiologist [32]. The transition from TAE to chemoembolization (TACE) seeks to not only embolize feeding vessels but also introduce high local concentrations of cytotoxic chemotherapy within the target lesions, and TACE is currently one of the available first-line liver-directed therapies for patients with unresectable, multifocal HCC. 

For conventional TACE (cTACE), chemotherapy such as doxorubicin is mixed into a lipiodol solution with an embolizing agent and injected into the tumor. Drug-eluting bead TACE (DEB-TACE) offers longer intratumor exposure and reduced systemic concentrations of doxorubicin afforded by the beads’ high binding affinity to doxorubicin [33]. It is currently unclear whether or not the formulations of DEB-TACE offer improved oncologic outcomes. A single small retrospective study of 71 consecutive patients treated over the span of 10 years by Dhanesekaran et al. showed a doubling of median overall survival (610 days vs. 284 days) when using DEB-TACE versus cTACE, though this was a single institution, non-randomized study spanning different technological eras in imaging capability (1998–2008) [34]. Larger studies (PRECISION V) were unable to demonstrate differences in survival, complete response rate, or objective response rates between DEB-TACE and cTACE. However, for patients with Child-Pugh B, ECOG 1, bilobar disease, and recurrent disease, DEB-TACE showed a significant increase in objective response [35]. 

It should be noted that, in contrast to TARE and SABR (stereotactic ablative body radiation, discussed below), TACE is not considered an ablative treatment, and durable tumor response rates are highly variable, even with serial treatments. To date, there is no prospective randomized evidence suggesting improved survival with TACE over simple transarterial embolization [36,37,38,39]. To this end, there are no standardized guidelines, except expert consensus, indicating the optimal embolizing agent, chemotherapy agent, amenable tumor sizes, target volumes (lobe, segment, or subsegment), or an allowable number of TACE procedures per lesion, and practice patterns vary widely both regionally and internationally [33]. Nonetheless, while TACE is not an ablative management strategy, its cytoreductive effects have led to ~20% improvements in 1- and 2-year overall survival when compared to best supportive care [40,41]. 

The future role of TACE remains unclear when considered alongside more modern liver-directed therapies such as TARE and SABR, which are able to provide ablative doses of focal radiotherapy, the latter having recently demonstrated improved overall survival when added to sorafenib (RTOG 1112, not published yet). When TACE was used in combination with SABR, there appeared to be higher control rates with planned combination therapy as opposed to TACE followed by salvage SABR when evaluated in a non-randomized fashion, establishing the hypothesis that RT and TACE may be synergistic [42,43]. This led to a Korean prospective randomized phase 2 study of TACE + RT (non-SABR technique) versus sorafenib in 90 patients with advanced tumors [44]. This study demonstrated that first-line therapy using TACE + RT in combination was well tolerated and had superior progression-free survival rates compared to sorafenib (87% vs. 34% at 12 weeks, *p* < 0.001), as well as improved survival. A recent study by Buckstein et al. at Mount Sinai demonstrated objective response rates of over 90% when TACE + SABR were used in combination therapy [45]. While the Yoon study of TACE + RT and the Buckstein study of TACE + SABR demonstrated favorable results of combination therapy, the results raise the subsequent question of whether or not TACE is needed when using ablative SABR, especially when biological doses near or exceeding 100 Gy biologically effective dose (BED) are used, which has demonstrated durable local control rates >80–90% in multiple disease sites (liver, lung, adrenal, others). The recently presented RTOG 1112 data showed improved OS with the addition of SABR to sorafenib alone, and further challenges the current utility of TACE in the advanced setting [46]. 

### 2.3. Transarterial Radioembolization (TARE)

The use of Y90 transarterial radioembolization (TARE) has become a highly utilized treatment of choice in the era of LDTs due to its ability to deliver ablative doses of radiation deposited intravascularly near the tumor site via feeding vessels and its versatility to simultaneously treat one or more lesions via either a radiation sub-segmentectomy, segmentectomy, or lobectomy. The beta decay of Y90 is well characterized and has a distinct dose-depth deposition, and to date, there are two major manufacturers (Sirtex^®^ and TheraSphere™), who respectively produce either resin particles or glass microspheres. Similar to TACE, TARE requires intravascular access via the hepatic arterial system, during which an interventional radiologist accesses the nearest feeding vessel supplying the tumor and, once confirmed via angiogram, injects Y90-embedded nanoparticles to deliver locally ablative doses of radiation. In contrast to TAE/TACE, TARE does not require embolization/coagulation and instead causes cell death via direct ionizing radiation emitted from Y90 nanoparticles. 

The current paradigm for activity selection in TARE relies primarily on two methods: the medical internal radiation dose (MIRD) model and the Partition Model. These models use the desired dose (Gy), fractional lung shunt, and estimated treated liver mass or tumor volume to calculate the desired injected activity (Gbq). As with all LDTs, local control is evaluated post-procedurally in the months following treatment using contrast-enhanced CT or MRI. Current estimates of per-lesion local control rates are difficult to pinpoint, though a multicenter study of segmentectomies in 102 patients with solitary HCCs reported a CR rate of 47% and a PR rate of 39%, and other studies report response rates of 40–42% [47,48,49]. The lack of high-quality long-term local control data for TARE is likely attributed to (1) its role as a relatively new treatment modality, and (2) high variance in its implementation across different institutions, ranging from partial Y90 hepatectomies to segmentectomies, and even sub-segmentectomies for single tumor ablation. 

For patients with advanced disease not suitable for resection or additional locoregionally directed therapies, systematic therapy is the primary standard of care therapy [50]. Previous large randomized prospective studies of TARE versus sorafenib demonstrated similar survival rates with the use of TARE for advanced disease and reported diminished rates of Grade 3+ adverse effects, such as gastrointestinal and dermatologic toxicity, suggesting some utility as an alternative treatment for patients unable to tolerate sorafenib but amenable to transarterial therapy [51,52]. Interestingly, the RTOG 1112 study of locally advanced disease is the first study to demonstrate improved survival with the addition of liver-directed therapy via external beam SABR to sorafenib (15.8 mo SABR + sorafenib vs. 12.3 mo sorafenib alone) despite the planned delay to start sorafenib due to SABR treatment. To date, TARE has not yet been compared to SABR with or without sorafenib in a prospective randomized fashion, and this remains a topic for future study as they both provide ablative-intent local radiation. As the field continues to evolve, there are newer first-line agents for systemic therapies, including tremelimumab with Durvalumab or Lenvatinib. These agents have yet to be reported with LDTs prospectively. 

The use of dimensional voxel-based dosimetric planning and post-treatment dosimetric verification of TARE is an emerging technology. Software packages are currently available (Varian Eclipse, Simplicit90Y, and MIM), which are capable of performing voxel-based dosimetry workflows to generate dose-volume histograms (DVHs) of gross tumor volumes (GTVs) as well as liver and lungs. These workflows have the advantage of improved dosimetric precision compared to conventional pre-treatment planning systems, such as the MIRD or the Partition model [53]. These models (as they currently stand) rudimentarily assume a uniform distribution of nanoparticles within a target volume and do not account for the reality of “hot” and “cold” spots due to heterogeneity of particle distribution and the short therapeutic range of β emission (~2.5 mm). Voxel-based dosimetry provides information such as the location of the maximum dose within the target volume, minimum dose delivered to the tumor, and mean target dose, as well as dose parameters for uninvolved liver parenchyma (liver minus GTV). In this way, Tc-MAA dosimetric techniques allow interventional radiologists to better assess three-dimensional treatment volumes and better select planned doses (GBq) to cover the tumor and assess the dose to the uninvolved liver parenchyma as well as other organs at risk. These techniques have the potential to select ideal candidates for which the intravascular dispersion of nanoparticles fully covers the target, as well as tumors that would be grossly underdosed (under-covered) with transarterial radiotherapy. The appeal of this workflow lies in the ability to alter planned treatments before the dose is delivered. Current limitations include reliance on multiple image registrations of diagnostic MRI, SPECT, CT, and/or PET, respiratory motion during image acquisition, blooming effects of PET and SPECT, as well as inherent differences in the physical characteristics of the nanoparticles (Tc-MAA, resin, spheres). These factors all add varying degrees of uncertainty and user dependence when using planning dosimetry workflows. 

Y90 PET-based post-treatment dosimetry techniques allow the treating team to evaluate the delivered dose as opposed to the planning dose. A post-injection PET scan fused to a diagnostic CT or MRI may be used to calculate the voxel-based dose distribution of the absorbed dose. Post-treatment dosimetry bears similar limitations in precision as Tc-MAA planning (above) but eliminates the variable of Tc-MAA as a surrogate for Y90. Another limitation of post-treatment dosimetry is that dose modifications cannot be made after injection of the payload (i.e., the dose cannot be withdrawn or redistributed), even in the event that a geometric miss of gross tumor volume is confirmed. Early studies of 3D dosimetry have suggested that there is a positive signal of dose-volume relationship between tumor dose and tumor control around 205–225 Gy [54,55,56]. DOSPHERE-01 is a prospective randomized study comparing standard MIRD (120 ± 20 Gy) vs. personalized dosimetry (≥205 Gy targeted to the index lesion). Patients who had personalized dosimetry had a significantly better objective response (71% vs. 36%, *p* = 0.0074) [55]. Taken together, both pre-treatment and post-treatment voxel-based dosimetry are compelling areas of development and warrant further prospective validation. 

### 2.4. External Beam Radiation Therapy

The history of radiation therapy (RT) used in HCC could be traced back decades. Given respiratory motion, set-up challenges, and a lack of effective tracking and immobilization methods, initially, radiation oncologists needed to treat large volumes to adequately cover the primary liver tumor, or in many cases, the whole liver. This resulted in significant treatment-related toxicity, known as radiation-induced liver disease (RILD), and limited RT to a palliative role in HCC management [57]. RILD usually occurs about 8 weeks following RT, and most of the patients recover within 3–5 months [58]. Based on clinical presentations, RILD is classified as classic and nonclassic. In classic RILD, the symptoms include anicteric hepatomegaly, ascites, and elevated liver enzymes, while in nonclassic RILD, patients usually only have markedly elevated serum transaminases and jaundice [59]. RILD is a subacute pathologic process with the hallmark of veno-occlusive disease or venous congestion of the central portion of the liver lobule, which would cause hepatocyte atrophy and fibrosis [60,61]. The etiology of RILD is thought to be damage from RT to normal liver tissue, and the risk of RILD is closely related to the radiation dosage. The risk of RILD is approximately 5% for patients who received 28 Gy of 2 Gy daily fraction, while those who received 36 Gy of 2 Gy daily fraction had a risk of 50% of RILD [58,62,63,64]. Dawson et al. evaluated dose-volume tolerance for RILD using a modified normal tissue complication probability model. They studied 203 patients who had conformal liver RT and hepatic arterial floxuridine and found no RILD when the mean liver dose was less than 31 Gy [65]. With the advancement of technology, RT can be delivered in a much more precise and conformal way to achieve better normal tissue constraints, which significantly decreases the risk of RILD. 

Stereotactic ablative body radiotherapy (SABR), also known as stereotactic body radiation therapy (SBRT), is a type of RT that can precisely deliver a high dose of radiation to ablate tumor cells while causing minimal damage to the surrounding normal tissue. SABR typically requires five or fewer treatments with a much higher dose per treatment. Though the total dose is usually the same or even smaller in SABR compared with traditional RT, the higher dose per fraction has a different biological effect. Of note, it is arbitrary to compare the total dose when comparing different RT fractionation schemes. The concept of BED is used for isoeffective dose fractionation calculations when trying to compare different doses and fractionation schemes. BED calculates the biologically effective dose by considering the total dose, dose per treatment, and tissue’s radiosensitivity (α/β ratio) [66,67,68]. The formula for BED calculation is: $BED=nd\left[1+\frac{d}{\frac{\alpha }{\beta }}\right];$ where *n* is the number of fractions, d is the dose per fraction, and α/β ratio is the number reflected by the tissue’s radiosensitivity (typically 10 for early-responding tissues and tumors and 3 for late-response ones). There are multiple websites or applications available for quick BED calculation [69,70]. With the same amount of total dose, a higher dose per treatment regimen usually has a higher BED. For instance, with an α/β ratio of 10, the BED for 50 Gy in 5 fractions (10 Gy per fraction) is 100 Gy, while that for 50 Gy in 25 fractions (2 Gy per fraction) is 60 Gy [68]. Based on this, SABR usually has a higher BED than traditional RT. Those high equivalent doses would have a much greater cell-killing effect given that the relation between radiation dose and cytotoxic effect is nonlinear [71]. Higher BED can also cause more DNA damage-based injury, and has antiangiogenic effects on endothelial cells, resulting in tumor vasculature damage, which leads to tumor hypoperfusion, hypoxia, and indirect cell death [71]. Moreover, studies have proven that SABR can induce immune cell activation, further enforcing the antitumor effect [72]. Besides those therapeutic advantages, SABR can reach better normal structure constraints compared to traditional RT [73,74]. This trait of SABR is pivotal for liver radiation since liver function preservation is critical in HCC patients. 

Multiple studies have proven SABR is efficacious as definitive therapy, salvage therapy, bridge to transplant, or palliative therapy in HCC patients. As with traditional RT, SABR is a non-invasive treatment modality. It can be delivered safely to patients who are not able to tolerate invasive procedures medically. Patients can return to normal activities right after treatment and have a lower risk of treatment-related side effects compared with other invasive LDTs. The main limitation of SABR is the estimated residual liver function post-treatment. Studies have recommended limiting single HCC lesion size to 15 cm, combined lesion size to less than 20 cm (up to 5 lesions), expected residual functional liver >700 cc, or liver functions in Child–Pugh A, B7, or B8 [75,76]. The newly developed albumin-bilirubin (ALBI) score might help predict a patient’s liver function and risk of RILD, thus facilitating candidate selection [77,78,79,80,81,82,83]. 

In the following, we compare SABR with other LDTs in detail. Of note, the choice of LDT should be based on multiple clinicopathological factors, including baseline liver function, performance status, HCC disease burden, patient preference, institutional availability, and expertise. A multidisciplinary discussion is highly recommended for treatment recommendations. 

## 3. Comparison of SABR with Other LDTs

### 3.1. SABR vs. RFA

RFA is a practical approach to destroying inoperable primary or metastatic liver tumors by precisely delivering high-frequency alternating electrical current to the tumor site. It is recommended as the first treatment option for HCC patients with very early-stage or early-stage disease who are not candidates for transplant [19]. Microwave ablation is another ablation modality used in HCC management. Data has shown that microwave ablation is equivalent to RFA in terms of overall survival, local recurrence, and complication rates [84,85]. Besides RFA and microwave ablation, SABR has also been reported as an effective ablative option for patients with small HCC [86,87,88]. Park et al. followed 290 HCC patients with a median tumor size of 1.7 cm; their 5-year LC and overall survival (OS) rates were 91.3% and 44.9% after SABR treatment, respectively [86]. Mathew et al. published similar data in which patients with smaller HCC (<3 cm) had better LC after SABR compared to RFA [87]. In a single-arm phase II clinical trial, Yoon et al. demonstrated that SABR could be a good alternative for patients with small HCCs (<5 cm) with either curative or salvage intent [88]. 

Given that both RFA and RT have been proven effective for small HCCs, many studies have compared the efficacy of these treatment modalities. So far, the most robust evidence comes from a Korean phase III randomized control trial [89]. The study recruited 144 recurrent/residual HCC patients with less than 3 cm and 2 lesions. Participants were randomly assigned to either proton-beam RT (PBT) or RFA. Due to this unique depth-dose feature, the proton beam can spare adjacent normal tissue and allow dose escalation to the tumor target, similar to SABR. With the non-inferiority design margin at 15%, they found PBT has non-inferior local progression-free survival (PFS) at 2 years (primary endpoint, PBT vs. RFA: 92.8% vs. 83.2% in the ITT population, 94.8% vs. 83.9% in the PP population) [89]. Aside from this clinical trial, there are also multiple retrospective studies directly comparing RT and RFA. The University of Michigan analyzed data from 224 nonmetastatic HCC patients with a median size of 2 cm using either RFA or SABR [90]. Freedom from local progression for patients treated with RFA was 83.6% and 80.2% for 1- and 2-year, respectively, while the numbers for SABR were 97.4% and 83.8%, respectively. Tumor size was a significant prognostic factor for RFA but not for SABR. In patients with a tumor larger than 2 cm, there was a decreased LC for RFA compared with SABR (HR 3.35, *p* = 0.025). Hara et al. also found that SABR has a comparable outcome compared with RFA for small HCC (≤3 cm) after analyzing data from 695 HCC patients [91]. The 3-year local recurrence rate was 5.3% vs. 12.9% (SABR vs. RFA), and the 3-year OS rate was 70.4% vs. 69.1% [91]. Similar results were found in the Asian population [92]. Besides those single-institution studies, some research based on public databases have also compared SABR with RFA [93,94]. Parikh et al. analyzed 440 patients with stage I or II HCC from the SEER-Medicare database and found that SABR and RFA have similar 90-day hospitalization and 1-year mortality rates [93]. Meta-analyses based on retrospective studies have demonstrated that SABR is an effective alternative to RFA, if not better [95,96,97]. Several ongoing clinical trials are comparing RFA and SABR for HCC, including a phase III randomized trial for HCC ≤5 cm (NCT03898921), a randomized trial for recurrent small HCC ≤5 cm (NCT04047173), and a phase III randomized non-inferiority trial for unresectable small HCC ≤3 cm (NCT05433701). The results of those trials could help clarify SABR efficacy in small HCCs, compared with RFA. 

### 3.2. SABR vs. TACE

SABR has been demonstrated as an alternative to TACE for treating HCC patients with specific conditions. However, high-quality data comparing these two modalities are lacking. A small pilot study by Nugent et al. of 29 patients randomized to either SABR or TACE demonstrated that SABR delivered to a mean dose of 45 Gy in 5 fractions had a re-treatment probability of 0% at 12 months versus 38.9% for TACE, favoring the ablative nature of external beam SABR [38], which was also supported by the updated data in 2020. In a single-institution retrospective study including 209 HCC patients, SABR was found to have better LC (1- and 2-year LC: SABR 97% and 91%; TACE 47% and 23%, *p* < 0.001) but similar OS (1- and 2-year OS: SABR 74.1% and 34.9%; TACE 75.3% and 54.9%, *p* = 0.21) [98]. Another single-center analysis revealed that SABR, even after prior HCC treatments, has equivalent 1-year LC and OS compared with TACE [99]. Similar results were presented in a multi-institutional retrospective propensity-matched analysis [100]. Su et al. analyzed data from 326 patients with inoperable BCLC-A HCC, of whom 167 patients had SABR with 28 to 50 Gy in 1 to 5 fractions and 159 patients underwent TACE [100]. The SABR group had improved LC (SABR vs. TACE, 1-, 3-, and 5-year: 86.8%, 62.5%, 56.9% vs. 69.3%, 53.3%, 36.6%, *p* = 0.0047), intrahepatic control (1-, 3-, and 5-year: 77.3%, 45.9%, 42.4% vs. 57.3%, 34.1%, 17.7%, *p* = 0.003), and PFS (1-, 3-, and 5-year: 63.4%, 35.9%, 27.5% vs. 53.5%, 27.4%, 14.2%, *p* = 0.049) [100]. The OS was comparable between two groups (1-, 3-, and 5-year: 85.7%, 65.1%, 62.8% vs. 83.6%, 61.0%, 50.4%, *p* = 0.29) [100]. SABR was associated with better local and intrahepatic control in multivariable analysis (HR = 1.59; 95% CI: 1.03–2.47; *p* = 0.04; HR = 1.61; 95% CI: 1.13–2.29; *p* = 0.009, respectively) [100]. 

Proton beam therapy is another external radiation modality being gradually tested in HCC management. As a charged particle, protons can penetrate a certain depth in tissues and deposit the majority of energy at the site of “Bragg Peak”. Due to this unique depth-dose feature, the proton beam can spare adjacent normal tissue and allow dose escalation to the tumor target, similar to SABR. A randomized clinical trial compared proton beam radiation therapy to TACE (NCT00857805) [101]. The interim analysis reported in 2016, including 69 available subjects, revealed similar OS rates on both arms (2-year OS 59%) [101]. The 2-year LC and PFS favor the proton beam group (LC: proton beam RT vs. TACE: 88% vs. 45%, *p* = 0.06, PFS: 48% vs. 31%, *p* = 0.06) [101]. The trial has completed enrollment, and the final results have not been published yet (NCT00857805). 

Multiple clinical trials are directly comparing RT, mainly SABR, with TACE. Besides the aforementioned trials (NCT00857805, NCT02182687, NCT03960008), there is another trial comparing SABR to TACE (NCT03338647), and a trial for RT vs. TACE in postoperative HCC (NCT02125396). Moreover, trials are exploring the efficacy of the combination of TACE and RT (NCT03895359, NCT02513199, NCT02794337), and re-TACE vs. SABR in post-TACE HCC (NCT02921139, NCT02762266). The ongoing IAEA E33036 study by the International Atomic Energy Agency is a large prospective randomized multinational study seeking to evaluate the non-inferiority of SABR when compared to TACE [102]. We will have better insights when making decisions between SABR and TACE with evidence from these trials. 

### 3.3. SABR vs. TARE 

TARE is another LDT by targeted delivery of high-dose radiation, Y90 microspheres, to the liver tumor via the hepatic artery. Growing evidence supports TARE efficacy in all stages of HCC. A prospective cohort study including 1000 HCC patients who underwent TARE over 15 years demonstrated favorable outcomes of TARE in all BCLC stages [103]. The censored OS was defined as from the first TARE until death, the last follow-up, or curative therapy. In CP A patients, the median censored OS for BCLC A, B, and C disease were 47.3, 25.0, and 15.0 months, respectively. The median censored OS for BCLC A, B, and C diseases in CP B patients was 27.0, 15.0, and 8.0 months, respectively [103]. Based on those results, Northwestern adopted TARE as the first-line transarterial LDT for HCC patients [103,104]. The following LEGACY study, published in 2021, further explored TARE efficacy in HCC patients. The study retrospectively analyzed data from 162 patients with tumors ≤8 cm from three medical centers [105]. TARE was used as neoadjuvant therapy for transplantation or resection (21% and 6.8%, respectively) or as primary treatment for others (72.2%). The objective response rate was 88.3%, with 62.2% of patients having a duration of response of at least 6 months. With a median follow-up of 29.9 months, the 3-year OS was 86.6% for all patients, and 92.8% for neoadjuvant patients who had subsequent resection or transplantation [105]. Higher-level evidence also supports TARE as an effective treatment for advanced HCC. In the randomized controlled phase III trial SARAH, Y90 was compared with sorafenib in locally advanced, inoperable HCC [51]. Here, 467 patients were randomly assigned. With a median follow-up of around 28 months (27.9 months in the Y90 group, 28.1 months in the sorafenib group), Y90-resin microsphere radioembolization demonstrated a comparable OS (median OS, Y90 vs. sorafenib: 8.0 vs. 9.9 months, *p* = 0.18) with fewer grade ≥3 treatment-related adverse events. Similar findings were presented in the SIRveNIB trial, a phase III open-label clinical trial comparing Y90 versus sorafenib for locally advanced HCC [52]. A total of 360 HCC patients from the Asia-Pacific region were enrolled. Y90-resin microsphere radioembolization has similar efficacy with no significant difference in OS (median OS, Y90 vs. sorafenib: 8.8 vs. 10.0 months, *p* = 0.36) [52]. Fewer patients in the Y90 group had severe adverse events (20.8% vs. 35.2%) [52]. Though SARAH and SIRveNIB did not prove that Y90 is superior to sorafenib, they did validate that TARE is a good treatment option for locally advanced HCC patients. The NRG/RTOG 1112 trial compares sorafenib versus SABR followed by sorafenib in new or recurrent HCC unsuitable for resection, transplant, ablation, or TACE, as discussed above. 

There is currently very limited data comparing Y90 TARE to other liver-directed therapies such as TACE, RFA, or SABR. In the only randomized controlled study of TARE versus TACE to date, albeit a small study of 45 patients [106], TARE was able to demonstrate a dramatic lengthening of time to progression (more than 26 months) versus TACE (6.8 months), with similar overall survival (~18 months) and highlights the relatively ablative nature of TARE when compared to chemoembolization. There are currently no additional published randomized controlled trials comparing the efficacy of TARE versus TACE in either the unresectable or resectable setting, though multiple meta-analyses have been performed, leading to varying and sometimes opposing conclusions [107,108,109]. As to data comparing TARE with SABR, a retrospective study including 87 patients reported comparable LC in the SABR and TARE groups (1-year LC, SABR vs. Y90, 87% vs. 89%, *p* = 0.76) [110]. Patients treated with SABR were more likely to have prior LDT, larger tumors, and higher tumor stages [110]. Some studies explored the role of SABR after extensive use of other local therapies, including Y90, and demonstrated that SABR could treat those patients safely with favorable outcomes (abstracts only) [111,112]. Though data is limited, evidence emerging in the near future might shine a light on the field. Two randomized clinical trials are ongoing. The first one is a single-site pilot study evaluating the efficacy of Y90 and SABR for solitary early-stage (≤3 cm) HCC (recruiting, NCT04235660). The other is a phase II trial exploring Y-90 segmentectomy versus SABR in treating inoperable liver cancer (not recruiting yet, NCT05157451). Besides those head-to-head comparison trials, one study is investigating the combination of Y90 followed by SABR in unresectable HCC (NCT04518748), which might offer a new pathway for selected HCC patients. 

### 3.4. SABR in HCC with MVI

Advanced HCC is frequently associated with macrovascular invasion (MVI) of major vessels, including the portal vein and hepatic vein [112,113,114,115]. Among them, portal vein tumor thrombus (PVTT) is the most common pattern [112,113,116]. MVI is a significant negative prognostic factor, with a median OS of about 2–5 months without treatment and about 6 months with the best supportive care [115,117,118,119,120]. Treatment for patients with MVI is challenging due to the extent of disease and liver dysfunction, even decompensation. The extension of the tumor to the vasculature facilitates tumor spread both intrahepatically and extrahepatically. MVI could impair liver function by reducing liver perfusion, directly reducing inflow in PVTT, and/or elevating sinusoidal pressure in hepatic vein invasion [121]. Current guidelines recommend systematic therapy as the first-line treatment for patients with HCC and MVI [15,19,122,123]. However, several local treatment modalities have been tested in this clinical scenario, including surgical resection, TACE, TARE, SABR, RT, or combinations [49,52,124,125]. 

RT has been reported as an effective LDT for HCC with MVI. In a retrospective cohort including 128 HCC and MVI patients who underwent SABR, Munoz-Schuffenegger et al. found that SABR has excellent LC (1-year 87%) and OS (median OS 18.3 months) [126]. Significantly, for those who received sorafenib after SABR, the median OS could prolong to 37.9 months. Besides, 27% of patients had a more than 2-point decline in CP score 3 months after SABR [126]. Another cohort study with 70 HCC and PVTT patients reported similar results [127]. A randomized multicenter clinical trial compared the efficacy of neoadjuvant RT followed by surgery versus surgery alone for patients with resectable HCC and PVTT [128]. A total of 164 eligible patients were randomized to either RT (18 Gy in 6 fractions to PVTT using 3D-RT) with surgery 4 weeks later or surgery alone within 1 week after randomization. With a median follow-up of 15.2 months, OS was found to be significantly better in the RT group (1-year OS: RT + surgery vs. surgery: 75.2% vs. 43.1%), as well as disease-free survival (33.0% vs. 14.9%). Multivariable analyses showed that neoadjuvant RT could significantly reduce HCC-related mortality and recurrence rates (HR 0.35 and 0.45, respectively, both *p* < 0.001) [128]. Another single-center randomized trial was designed to test SABR in the adjuvant setting for HCC with MVI [129]. A total of 76 patients with MVI-positive disease who had marginal resection were randomly assigned to adjuvant SABR (35 Gy in 5 daily fractions) vs. observation. With a median follow-up of 55 months, patients in the adjuvant SABR group had better disease-free survival (1-, 3-, and 5-year SABR vs. observation, 92.1%, 65.8%, 56.1% vs. 76.3%, 36.8%, 26.3%, *p* = 0.005) but similar OS (1-, 3-, 5-year: 100%, 89.5%, 75.0% vs. 100.0%, 68.4%, 53.7%) [129]. No grade ≥3 adverse events were observed in the SABR group [129]. Of note, the aforesaid SIRveNIB and SARAH trials also included a portion of MVI patients (SIRveNIB: 30% of all participants; SARAH: 63% of patients in the TARE group, 58% in the sorafenib group) [51,52]. Both trials demonstrated that TARE is as effective as sorafenib for locally advanced HCC with or without MVI.

RT is also efficacious when combined with other LDT in treating HCC with MVI. One randomized clinical trial tested the efficacy of the combination of TACE and RT versus sorafenib in HCC patients with MVI [44]. Here, 90 patients with liver-confined disease were equally assigned to either the sorafenib group (receiving sorafenib 400 mg twice daily) or the TACE-RT group (TACE every 6 weeks with RT starting within 3 weeks after first TACE, maximum 45 Gy in 2.5–3 Gy per fraction). The TACE-RT group demonstrated a higher 12-week PFS (TACE-RT vs. sorafenib: 86.7% vs. 34.3%, *p* < 0.001) and prolonged median OS (55.0 vs. 43.0 weeks, *p* = 0.04) [44]. 

### 3.5. SABR as Bridge Therapy to Transplant

SABR is efficacious as a bridge therapy to transplant compared with other LDTs. Mohamed et al. reported retrospective data from 60 patients using SABR (*n* = 24), TACE (*n* = 37), RFA (*n* = 9), or Y90 (*n* = 9) as a bridge to transplant (79 bridge therapies in total) [130]. The median time to transplant after bridge therapy was 7.4 months. Pathologic complete response was 41%, 28.5%, 60%, and 75% for TACE, SABR, RFA, and Y90, respectively [130]. SABR and Y90 showed significantly less grade ≥3 acute toxicity [130]. Another retrospective study from the University of Toronto analyzed a cohort of 379 HCC patients who underwent bridge therapy (SABR 36 cases, TACE 99 cases, RFA 244 cases) [131]. Postoperative complications were similar and no significant survival difference among groups. Actuarial 1-, 3-, and 5-year survival after listing were 83%, 61%, and 61% for SABR, 86%, 61%, and 56% for TACE, and 86%, 72%, and 61% for RFA (*p* = 0.4), respectively [131]. The 1-, 3-, and 5-year OS from the time of transplant were 83%, 75%, and 75% for SABR, 96%, 75%, and 69% for TACE, and 95%, 81%, and 73% for RFA (*p* = 0.7), respectively [131]. Data from clinical trials, though limited, also supports RT (SABR) as a good bridge therapy modality. A phase II randomized clinical trial compared SABR with TACE as a bridging therapy for HCC patients undergoing transplants [132]. Preliminary analysis from 50 patients (21 patients in the SABR group, 29 patients in the TACE group) revealed that SABR is as effective as TACE with potentially less toxicity [132]. The trial has completed recruitment (a total of 60 patients, NCT02182687), and a following phase III multi-center clinical trial is ongoing (NCT03960008).

## 4. MR-Linac SABR

Conventional RT is delivered with a computed tomography (CT)-based system. The CT-guided RT has many inherent limitations regarding precise planning and delivery. The magnetic resonance (MR)-guided RT (MRgRT) system was recently introduced to the field with the hope of overcoming these limitations [133,134,135,136]. By combining MR imaging with standard linear accelerators (MR-Linac) or Cobalt-60, the MRgRT can provide a better anatomic target definition and accurate motion management solution without external markers or implanted fiducials [133,134,135,136]. With high soft-tissue contrast, MRgRT could improve target volume delineation, better differentiating those targets close to or invading adjacent structures. The integrated on-table plan adaptation in MRgRT can help further reduce margins, improve organ at-risk sparing, and accommodate anatomical and physiological changes throughout RT. The continuous MR imaging system enables real-time gated treatment delivery, which could mitigate motion impact and facilitate smaller margins [134,135,136]. Overall, MRgRT has been proven to improve the therapeutic index of RT, which makes it an excellent treatment modality for HCC patients.

**Figure 1:**
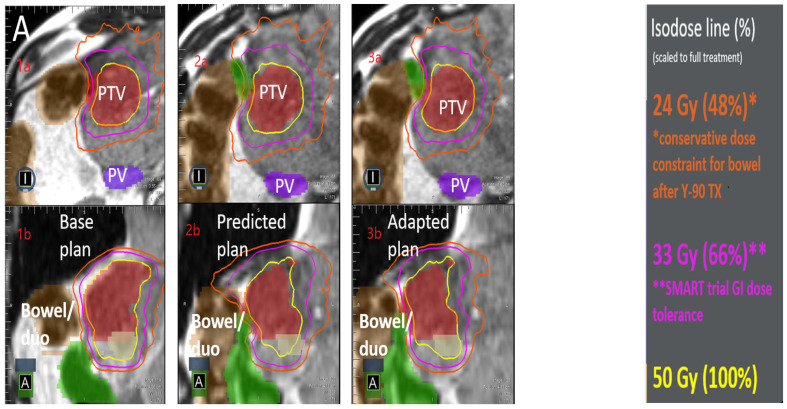

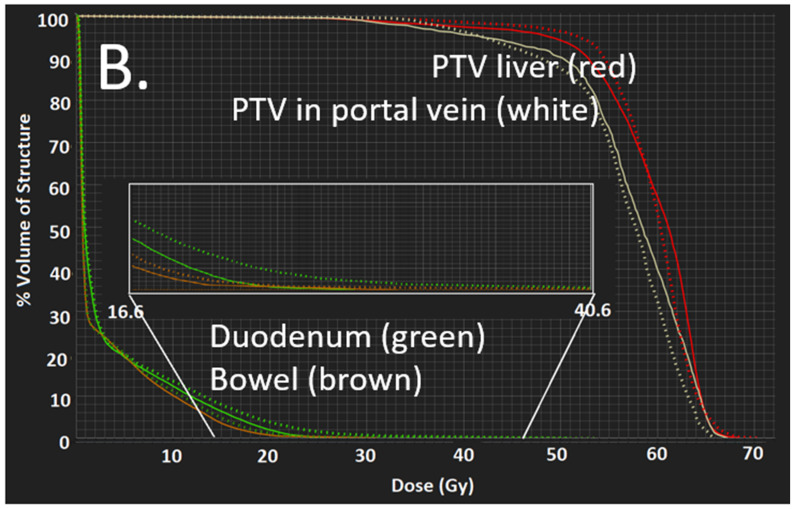
MRgRT treatment for tumors abutting very mobile organs such as GI structures. (**A**): Dose distributions from: left panel (**A1**): base plan in axial plane (**A1a**) and coronal plane (**A1b**), middle panel (**A2**): non-adaptive plan, i.e., what would be delivered with standard SABR, in axial plane (**A2a**) and coronal plane (**A2b**), and right panel (**A3**): MRgRT -based adaptive plan, in axial plane (**A3a**) and coronal plane (**A3b**). The isodose values are shown on the right most panel, with the orange and pink lines representing doses that would likely result in patient GI toxicity. (**B**): Radiation dose comparison for the adapted (solid) vs. standard (dash) treatment in (**A**), with an insert showing the higher dose to the duodenum (green) and bowel (brown) that would have been delivered in a conventional, non-adapted treatment.

Currently, two MR-linac systems are available in the market: the ViewRay MRIdian Linac and Elekta Unity MR-Linac [137]. The concept of stereotactic MRI-guided on-table adaptive radiotherapy (SMART) was brought forward after MR-Linac was recently popularized in the field [138,139,140]. SMART combines the MRgRT with SABR, and further expands the advantages of both. The workflow for MRgRT is institution-specific and patient-specific based on treatment goals. It usually requires sessions similar to CT-based RT, including simulation, planning, and treatment setup. The extra unique sessions, particularly online adaptation, on-table plan evaluation, tracking preview, and gated delivery [137,141,142] greatly enhances the efficacy of radiation therapy and minimizes radiation damage to nearby organs at risk. Figure 1 shows a treatment for a patient using MRIdian-based MRgRT with HCC abutting the bowel with portal vein thrombus, as well as prior TARE radiation right hepatectomy. In the base plan created a few days before treatment (Figure 1A(1a,1b)), isotoxic doses to GI structures (orange and pink isodose lines) were purposely curved away from GI structures (duodenum in green and bowel in brown). However, upon imaging directly prior to the treatment, both GI structures had shifted so close to the tumor that they overlapped with the region that would have received full prescription dose (Figure 1A(1a,2b)). In normal Linac-based SABR treatments, this could have caused severe toxicity to the patient. This is, in fact, why doses in conventional, non-adaptive SABR treatments are prescribed routinely to non-curative doses of 35 Gy when the tumor is located close to sensitive organs at risk. For this patient, adaptation with MRgRT allowed a curative dose to be delivered to the majority of the tumor while reducing the dose delivered to the bowel and duodenum up to 50% relative to the non-adaptive plan (Figure 1A(1c,2c),B). This capability for dose escalation to a curative dose while still limiting tissue toxicity is the reason for the SMART trial and it highlights the greatest advantage of MRgRT, with one-year LC at 87.8% [143].

Though experience with MRgRT is still in its infancy, there is growing evidence supporting a role in HCC management. A multi-institutional study explored the MRgRT efficacy in treating liver tumors, including 6 HCC patients, 2 cholangiocarcinoma patients, and 18 metastatic liver lesions. Small treatment volumes were achieved using MRgRT, and no grade 4 or more significant gastrointestinal toxicities were observed. With a median follow-up of 21.2 months, the freedom from local progression rate was 80.4% for all enrolled patients, with 100% for HCC cases. The 1- and 2-year OS were 69% and 60%, respectively [144]. Similar results were reported in another study with 15 liver lesions from cholangiocarcinoma or metastatic diseases [145] and a study including 2 HCC and 18 metastatic liver tumors [146]. A report from a 2.5-year MRgRT experience included 316 patients, among which 21% were abdominal cases (18 liver sites treated with adaptative RT) [147]. Patients were selected for various indications, including improved soft tissue contrast, cine gating, online adaptation, and, most commonly, a combination of the above. MRgRT proved to offer the aforementioned advantages [147]. Hal et al. reported a series of 10 abdominal cases using MRgRT (2 HCC and 4 live metastasis patients), in which all patients tolerated the treatment well with good outcomes [148]. Apart from those retrospective data, there is also preliminary prospective evidence supporting MRgRT/SMART role. A phase I trial studied the feasibility, safety, and efficacy of MRgRT/SMART in liver tumors [149]. Twenty patients (8 primaries, 12 metastatic) with 25 liver tumors were treated with MRgRT/SMART to a median dose of 54 Gy in a median of 3 fractions. The estimated 1- and 2-year LC were 94.7% and 79.6%, respectively; the 2-year estimated OS was 50.7% [149]. MRgRT/SMART presented great safety profiles without any severe acute toxicities [149].

MRgRT has also been applied for HCC with PVTT. In a retrospective cohort, 12 cases of unresectable HCC and PVTT were treated with either MRgRT (50 Gy in 10 fractions) or SMART (36–50 Gy in 4–5 fractions). Ten patients had an objective response, and 1-year intrahepatic control rate was 48.9%. No grade ≥3 toxicity was observed [150]. 

There are several challenges of MRgRT. It is resource intensive for a radiation oncology department as it is a newer modality of delivering radiation, requiring increased staffing, treatment planning, and upfront capital costs. MRgRT requires extra training for not only physicians but also dosimetrists, physicists, and therapists who work behind the scenes to develop the radiation plan. MRgRT sessions take more time on the treatment machine than traditional SBRT due to more complicated patient setup, online adaptation plan evaluation, gated delivery, and slower dose delivery. The prolonged on-table time could lead to possible treatment failure or re-planning due to patient intolerance. From a patients’ perspective, MRgRT might be less comfortable than traditional SBRT, with longer on-table time, lower room temperature to keep the MRI scanner and Linac accelerator functioning optimally, and the need to consistently hold their breath during a long treatment [146]. The gated-delivery system requires patients to adjust their breathing to center the target in the field, which might be difficult for patients with underlying respiratory diseases, like COPD. For patients with severe claustrophobia, MRgRT should be applied cautiously. Patients with implanted ferromagnetic and/or electrical devices have to be vetted prior to treatment. 

Lastly, MRgRT treatment delivery has overcome significant challenges by combining MRI imaging with a linear accelerator, where traditionally the combination would lead to significant radiation beam distortions. In addition, the movement of the linear accelerator gantry induces electric currents in the system. These currents can cause artifacts on MRI images used for tracking, thus limiting the manner in which doses can be delivered [151]. This limitation can result in less ideal dose distribution in some circumstances [151,152,153]. Despite this, however, the capability to adapt a plan for patient anatomical changes and the ability to track motion during treatment make MRgRT invaluable for specific tumor locations.

## 5. Conclusions

LDTs for HCC have proven efficacious and feasible, with a good safety profile (selected studies in Table 1). Among all LDTs, thermal ablation, TARE, and SABR can offer potentially curative treatment. SABR is an evolving modality applicable across all HCC spectrums, including as a bridge to transplant, local treatment for BCLC-A and B disease, and HCC with MVI. As patients with HCC await transplant, there may be a need for multiple bridging therapies, and further understanding regarding how retreatment affects liver function is needed. SABR can also be applied as an alternative treatment to TACE, RFA, or salvage treatment for recurrent tumors. Studies examining the role of TARE versus SABR are in their infancy, and more data is needed to discern when to employ either LDT. MRgRT is another non-invasive method to deliver SABR that further improves therapeutic dose while minimizing normal tissue/organ exposure, providing a new tool in the armamentarium of LDT. Ongoing clinical trials could help us better understand each LDT’s advantages and limitations, thus better tailoring treatment decisions. Multidisciplinary discussion is essential for personalized recommendations for HCC patients.

## Figures and Tables

**Table 1:** Selected LDT related studies.

Author	Study	Year	Patients	Modality	Outcomes
Park et al. [86]	Retrospective	2020	290	SABR	5 y OS 44.9%, 5 y LC 91.3%
Mathew et al. [87]	Retrospective	2020	436	SABR	5 y OS 77.3%, 3 y LR 13.3%
Yoon et al. [88]	Phase II single arm	2020	50	SABR	5 y OS 77.6%, 5 y LC 97.1%
Kim et al. [89]	Phase III	2021	144	PBT vs. RFA	2 y LPFS 94.8% vs. 83.9% (per protocol population), 92.8% vs. 83.2% (intention to treat population), *p* < 0.001
Wahl et al. [90]	Retrospective	2016	224	SABR vs. RFA	2 y FFLP 83.8% vs. 80.2%; 2 y OS 46.3% vs. 52.9% (NS); RFA vs. SABR LR hazard ratio 3.94, *p* = 0.002
Hara et al. [91]	Retrospective	2019	231	SABR vs. RFA	3 y LC 5.3% vs. 12.9%, *p* < 0.01; 3 y OS 69.1% vs. 70.4%, *p* = 0.86
Kim et al. [92]	Retrospective	2020	2064	SABR vs. RFA	3 y LR 21.2% vs. 27.9%, *p* < 0.001
Rajyagaru et al. [94]	Retrospective (National Cancer Database)	2018	3980	SABR vs. RFA	5 y OS 19.3% vs. 29.8%, *p* < 0.001
Pan et al. [95]	Retrospective meta-analysis	2020	2732	SABR vs. RFA	3 y LC OR 0.54, *p* = 0.002 (favor SABR); 2 y OS OR 1.66 *p* < 0.00001 (favor RFA)
Lee et al. [96]	Retrospective meta-analysis	2020	2238	SABR vs. RFA	2 y LC for HCC and mets 83.8% vs. 71.8%, *p* = 0.024; 2 y LC for HCC only 84.5% vs. 79.5%, *p* = 0.431
Wang et al. [97]	Retrospective meta-analysis	2020	7928	SABR vs. RFA	OS pooled HR 1.09, *p* = 0.63; LC pooled HR tumors > 2 cm 0.42, *p* = 0.003 (favor SBRT); pooled HR tumors ≤ 2 cm 0.56, *p* = 0.17
Sapir et al. [98]	Retrospective	2018	209	SABR vs. TACE	2 y LC: 91% vs. 23%, *p* < 0.001, 2 y OS 34.9% vs. 54.9%, *p* = 0.21
Bettinger et al. [99]	Retrospective	2018	402	SABR vs. TACE	1 y LC 84.8% vs. 74.4%, *p* = 0.146; median OS 9 vs. 17 mo, *p* = 0.016. Matched cohort median OS 9 vs. 11 mo, *p* = 0.989
Su et al. [100]	Retrospective	2020	326	SABR vs. TACE	5 y OS 62.8% vs. 50.4%, *p* = 0.29; 5 y PFS 27.5% vs. 14.2%, *p* = 0.049; 5 y LC: 56.9% vs. 36.6%, *p* = 0.0047, 5 y intrahepatic control 42.4% vs. 17.7%, *p* = 0.003
Bush et al. [101]	RCT (interim analysis)	2016	69	PBT vs. TACE	PCR 25% vs. 10% (*p* = 0.38); 2 y OS 59% both groups; 2 y LC 88% vs. 45%, *p* = 0.06; 2 y PFS 48% vs. 31%, *p* = 0.06
Salem et al. [105]	Retrospective	2021	162	Y90	Objective response rate 88.3%, 3 y OS 86.6%
SARAH [51]	RCT phase III	2017	467	Y90 vs. sorafenib	Median OS 8 vs. 9.9 mo, *p* = 0.18
SIRveNIB [52]	RCT phase III	2018	360	Y90 vs. sorafenib	Median OS 8.8 vs. 10 mo, *p* = 0.36
NRG/RTOG 1112 [46]	RCT (interim analysis)	2022	193	SABR + sorafenib vs. sorafenib	Median OS 15.8 vs. 12.3 mo, *p* = 0.0554; median PFS 9.2 vs. 5.5 mo, *p* = 0.0001
Mohamed et al. [130]	Retrospective	2016	60	SABR, Y90, TACE, and RFA prior to transplant	PCR 28.5%, 75%, 41%, 60%
deBettencourt et al. [110]	Retrospective	2021	87	SABR vs. Y90	1 y LC 87% vs. 89%, *p* = 0.76
Munoz-Schuffenegger et al. [126]	Retrospective	2021	128	SABR in HCC with MVI	1 y LC 87.4%, median OS 18.3 mo
Shui et al. [127]	Retrospective	2018	70	SABR in HCC with PVTT	Median OS 10 mo
Wei et al. [128]	RCT	2019	164	Neoadjuvant RT + hepatectomy vs. hepatectomy alone	2 y OS 27.4% vs. 9.3%, *p* < 0.001; 2 y DFS 13.3% vs. 3.3%, *p* < 0.001
Shi et al. [129]	RCT	2022	76	Surgery + adjuvant SABR vs. surgery alone	5 y DFS 65.1% vs. 26.3%, *p* = 0.005; 5 y OS 75% vs. 53.7%, 0.053
Yoon et al. [44]	RCT	2018	90	TACE + RT vs. sorafenib in HCC with MVI	12 wk PFS 86.7% vs. 34.3%, *p* < 0.001; OS 55 vs. 43 wks, *p* = 0.04
Sapisochin et al. [131]	Retrospective	2017	379	SABR, TACE, RFA bridge to transplant	5 y survival from transplant listing 61% vs. 56% vs. 61%, *p* = 0.4
Nugent et al. [132]	Phase II (abstract)	2020	60	SABR vs. TACE bridge to transplant	Time to recurrent or residual disease 10.4 vs. 9.2 mo, *p* value not available
Rosenberg et al. [144]	Retrospective	2018	26 (6 HCC, 2 cholangiocarcinoma, 18 metastases)	MRgRT	2 y OS 60%; PFS 35%; FFLP for HCC 100%; PFS for HCC 33%
Rogowski et al. [145]	Retrospective	2021	11 (2 cholangio-carcinoma, 9 metastases)	MRgRT	5 mo local failure 0%
Weykamp et al. [146]	Prospective observational	2021	20 (2 HCC, 18 metastases)	MRgRT	1 y LC 88.1%, 1 y OS 84.0%
Van Dams et al. [149]	Phase I	2021	20 (5 HCC, 3 cholangiocarcinoma, 12 metastases)	MRgRT	2 y LC 79.6%, 2 y OS 50.7%, median OS 29 mo
Lee et al. [150]	Retrospective	2022	12	MRgRT in HCC with PVTT	1 y intrahepatic control 48.9%, LC 83.3%

SABR, stereotactic ablative body radiation; PBT, proton-beam RT; RFA, radiofrequency ablation; TACE, transarterial chemoembolization; HCC, hepatocellular carcinoma; MVI, macrovascular invasion; PVTT, portal vein tumor thrombus; RT, radiation therapy; MRgRT, MR-guided adaptive radiation therapy; LC, local control; PFS, progression-free survival; LR, local recurrence; FFLP, freedom from local progression; OS, overall survival; HR, hazard ratio; PCR, pathological complete response; DFS, disease-free survival.

## Data Availability

Not applicable.
